# Dr. Francis Findlay

**Published:** 1913-01

**Authors:** 


Dr. Francis Findlay, Buffalo 1860, of Franklinville, N. Y., died at Parkville, Mo., Oct. 3, aged 78.

